# Zero Excess Sludge Production in a Thermophilic MBR System: Performance and Energy Consumption Evaluation

**DOI:** 10.1002/wer.70538

**Published:** 2026-08-14

**Authors:** Maria Cristina Collivignarelli, Stefano Bellazzi, Laura Maria Rita Calabria, Marco Baldi, Marco Sordi, Barbara Marianna Crotti, Alessandro Abbà

**Affiliations:** ^1^ University of Pavia: Università degli Studi di Pavia Pavia Italy; ^2^ Interdepartmental Centre for Water Research University of Pavia Pavia Italy; ^3^ Department of Chemistry University of Pavia Pavia Italy; ^4^ ASMia Srl—ASMortara SpA Mortara Italy; ^5^ Department of Civil, Environmental, Architectural Engineering and Mathematics University of Brescia Brescia Italy

**Keywords:** pure oxygen bioreactor, sludge minimization, thermophilic sludge treatment, ultrafiltration membranes, volatile solids removal

## Abstract

Water utilities are looking for creative solutions that can lower sludge output while maintaining process dependability and environmental compliance due to the increasing operational and regulatory difficulties related to managing sewage sludge. In order to minimize sludge, this study presents the first full‐scale implementation of a thermophilic pure‐oxygen biological process integrated with ceramic ultrafiltration (UF) membranes in Europe. The system uses thermophilic conditions (50°C–55°C) to treat thickened municipal sludge, and membrane separation ensures complete biomass retention. Long‐term monitoring (2024–2025) revealed high biodegradation performance, with VS removal exceeding 85% and very low biomass yields (0.01 kg VS kg^−1^ COD_removed). COD removal efficiencies were slightly lower due to the selective permeation of soluble organic matter, which is beneficially reused as an external carbon source for downstream denitrification. Oxygen‐ and energy‐based indicators confirmed that process efficiency was strongly influenced by membrane hydraulic performance, particularly membrane fouling, permeability decline, and the associated membrane maintenance requirements. Despite higher energy demand compared to conventional mesophilic processes, the substantial sludge reduction and recovery of soluble COD make the system economically competitive in a regulatory context where sludge disposal options are increasingly limited. The results demonstrate the robustness and sustainability of thermophilic UF‐based sludge reduction and highlight its potential as a strategic solution for modern wastewater treatment plants seeking resilient, circular, and regulation‐proof sludge management pathways.

## Summary

- Thermophilic pure‐oxygen treatment with UF membranes achieves > 85% volatile solids removal at full scale.
- Ceramic UF ensures complete biomass retention, enabling extremely low sludge yields and stable long‐term operation.
- Soluble COD permeation can be beneficially reused as an external carbon source for denitrification.
- Process efficiency and energy demand are strongly influenced by membrane fouling and hydraulic performance.
- Significant sludge reduction improves economic viability where sludge disposal options are limited.

## Graphical abstract

A full‐scale thermophilic pure‐oxygen process coupled with ceramic ultrafiltration achieves over 85% volatile solids removal and extremely low biomass yields, enabling substantial sludge reduction. Despite higher energy demand, the recovery of soluble COD and robust long‐term performance make this approach a sustainable and regulation‐resilient sludge management solution.

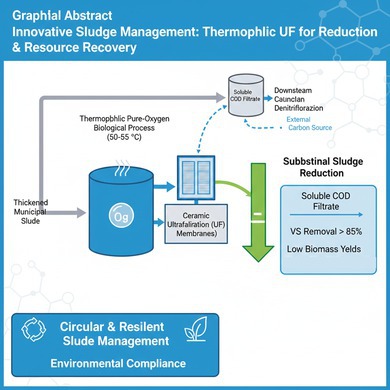

## 1 Introduction

The management and disposal of sewage sludge represent one of the most critical and complex challenges in the wastewater treatment sector. The increasing volumes of sludge generated—driven by population growth and the continuous expansion and upgrading of wastewater treatment plants—have intensified the need for sustainable and economically viable solutions for its valorization or final disposal (Kelessidis and Stasinakis 2012; Kacprzak et al. 2017). However, establishing reliable disposal pathways has become increasingly difficult due to a regulatory framework that is rapidly evolving and characterized by progressively stricter environmental and technical requirements.

Traditionally, sludge management has relied on a limited number of established pathways, including direct land application in agriculture, composting, co‐digestion and energy recovery, use in the production of soil amendments such as liming material (called defecation lime) from sludge, and, to a decreasing extent, landfilling and incineration (Kadam et al. 2024). However, establishing reliable disposal and recovery routes has become increasingly difficult due to a regulatory framework that is rapidly evolving and characterized by progressively stricter environmental and technical requirements.

Agricultural reuse, historically considered one of the most sustainable options due to its potential for nutrient recycling and soil conditioning, is increasingly constrained by regulatory limits on heavy metals, pathogens, organic micropollutants, and emerging contaminants such as microplastics, pharmaceuticals, and per‐ and polyfluoroalkyl substances (PFAS) (Pistocchi et al. 2019). Alternative recovery routes, such as the use of stabilized sludge in the production of liming material from sludge for agricultural purposes or soil remediation, as well as composting for horticultural and landscaping applications, are also facing growing scrutiny in terms of product safety, traceability, and long‐term soil pollutant accumulation effects (Ahmed et al. 2024). In parallel, thermal treatment options, including incineration, pyrolysis, and gasification, are gaining attention due to their potential for volume reduction, energy recovery, and pollutant destruction, albeit with significant economic and infrastructural implications (Samolada and Zabaniotou 2014).

At the European level, the recent revision of the Urban Wastewater Treatment Directive (EU Directive 2024/3019) and updates to the Fertilizing Products Regulation (EU 2019/1009) have introduced more stringent criteria for sludge intended for agricultural use, with particular emphasis on emerging contaminants (de Romph 2018).

Moreover, Regulation (EU) 2020/741 on water reuse adds further requirements to the management of treatment by‐products. The broader policy context shaped by the Waste Framework Directive (2008/98/EC, amended by Directive 2018/851) and the Landfill Directive (1999/31/EC) strengthens the push toward circular economy strategies and the reduction of landfill disposal, placing additional pressure on sludge management practices and encouraging processes oriented toward resource recovery and volume reduction (European Union 1999).

In Italy, the implementation of these European regulations intersects with a complex national landscape that includes Legislative Decree 152/2006, Legislative Decree 99/1992 regulating the agricultural use of sludge, and numerous regional provisions that impose even more restrictive limits for heavy metals, organic micropollutants, and hygienic parameters. This multilayered regulatory framework—compounded by recent court rulings and ministerial interpretations creates a fragmented scenario that complicates long‐term planning for wastewater utilities (Grassi 2019).

Within this regulatory and operational context, sludge minimization has increasingly become a strategic objective for wastewater treatment plants. Currently, the most widely implemented technologies for sludge reduction include anaerobic digestion, which combines volume reduction with biogas production and energy recovery (Oladejo et al. 2018); extended aeration and endogenous respiration processes; thermal hydrolysis pre‐treatment to enhance biodegradability; and mechanical and thermal drying systems aimed at reducing transportation and disposal costs (Nylen and Sheehan 2023). Emerging approaches, such as advanced oxidation processes, enzymatic treatments, and thermophilic biological processes, are being developed to further intensify sludge reduction while minimizing environmental impacts and improving overall process sustainability (Talreja et al. 2025).

At the same time, growing concern regarding emerging contaminants such as microplastics, pharmaceutical residues, and PFAS is accelerating the development of new risk assessment criteria and imposing additional regulatory and technological challenges. Within this context, the development and evaluation of innovative sludge treatment solutions have become essential to reduce environmental impacts, enhance process safety, and improve the overall sustainability of the integrated water cycle. This study contributes to this effort by presenting the implementation of the first plant in Italy and Europe designed for sludge minimization through a thermophilic biological process coupled with an ultrafiltration‐based solid–liquid separation system, offering new insight into future perspectives for a more resilient sludge management approach aligned with evolving regulatory requirements.

## 2 Materials and Methods

### 2.1 Process Description

**FIGURE 1:**
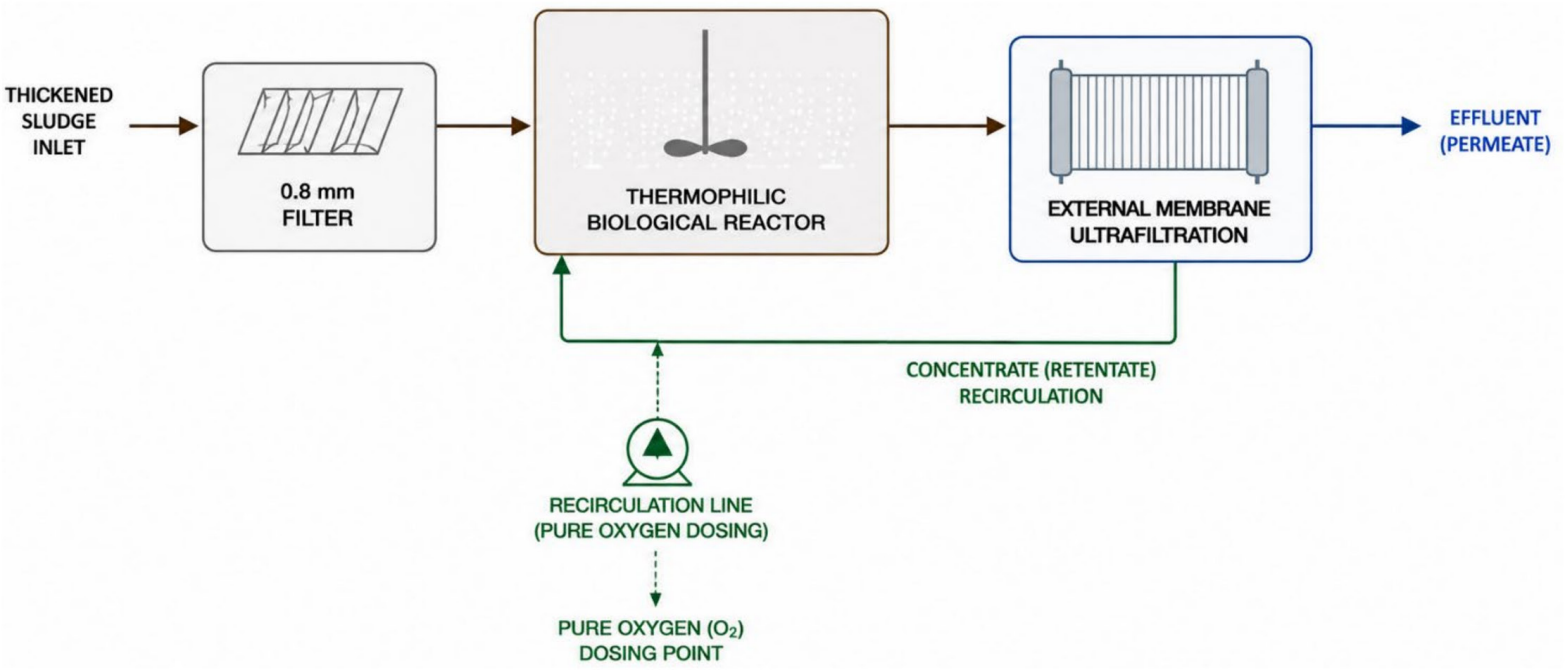
Thermophilic biological reactor operated with pure oxygen, integrated with a ceramic ultrafiltration (UF) membrane system for high‐efficiency solid–liquid separation.

The treatment platform investigated in this study consists of a thermophilic biological reactor operated under pure oxygen and coupled with a ceramic ultrafiltration (UF) membrane system for solid–liquid separation, Figure 1. The facility is located within a municipal wastewater treatment plant (capacity of 86.400 PE), where it processes the biological sludge produced by a conventional activated sludge line with a pre‐denitrification scheme. The sludge passes through a static thickener before going into the thermophilic unit to guarantee a steady and consistent feed concentration.

Prior to feeding the thermophilic line, the waste activated sludge is mechanically thickened using a static thickener in order to provide a stable and concentrated feed. During the monitoring period, the influent sludge flow rate ranged between approximately 10–30 m^3^ d^−1^, with an average value of 20 m^3^ d^−1^. The thickened sludge fed to the thermophilic reactor was characterized by COD concentrations generally ranging from 25,000 to 40,000 mg L^−1^, total solids (TS) concentrations of approximately [35–42] g L^−1^, and volatile solids (VS) concentrations of [20–30] g L^−1^. The average organic loading rate applied to the system was approximately 600 kg COD d^−1^.

The thermophilic reactor is operated at temperatures between 50°C and 55°C and is supplied with pure oxygen. The oxygen dosage is controlled to establish different oxygen concentration zones within the reactor, thus favoring high‐rate aerobic degradation in the fully aerated region and enabling oxygen‐limited conditions in localized micro‐zones that support nitrogen removal processes. Due to the typically poor settling characteristics of thermophilic biomass, the system relies entirely on membrane separation rather than clarification.

The reactor was operated with mixed liquor suspended solids (MLSS) concentrations generally maintained within the range of 60–120 g L^−1^, corresponding to mixed liquor volatile suspended solids (MLVSS) concentrations of approximately 40–60 g L^−1^. During the most stable operating phases, the average MLSS and MLVSS concentrations were approximately 80 and 50 g L^−1^, respectively. These elevated biomass concentrations were maintained through continuous membrane retention and retentate recirculation, allowing operation at extended sludge retention times (SRTs) and contributing to the very low observed biomass yield.

For the assessment of the plant's energy demand, electrical consumption is monitored using a Siemens SENTRON PAC3200 energy meter, which measures voltage, current, and active power. This device records the energy required for the operation of the feed, recirculation, and UF pumps, as well as the consumption associated with the sensors used for oxygen and temperature monitoring and control. In addition, the system manages the solenoid valves that regulate the membrane backwashing operations, typically activated every 10 min, using softened well water previously employed in the plant cooling system.

### 2.2 UF Membrane System

Solid–liquid separation is performed by a ceramic UF unit comprising three parallel vessels, each equipped with 99 tubular UF membranes. The membranes have a nominal molecular weight cut‐off of 300 kDa, and the system operates at transmembrane pressures ranging between 1 and 4 bar. The UF permeate is withdrawn continuously and represents the treated effluent, while the UF retentate is recirculated back to the thermophilic reactor to maintain the desired biomass concentration.

### 2.3 Reactor Operating Conditions

The thermophilic biological reactor has a working volume of approximately 200 m^3^ and was operated continuously under thermophilic conditions (50°C–55°C) with pure oxygen supply. The oxygen flow rate ranged between approximately 35–40 Nm^3^ h^−1^ at 9 bar, with a dissolved oxygen (DO) setpoint between 1.0 and 1.5 mg L^−1^; due to the high oxygen demand of the system, the reactor rarely reached the lower DO threshold and oxygen was therefore supplied for approximately 22 h d^−1^. Based on the average influent sludge flow rate, the hydraulic retention time (HRT) during the monitoring period was approximately 8–12 days, while the solids retention time (SRT) varied according to the operational strategy adopted to control membrane performance and biomass accumulation. Average SRT values were approximately 166.7 days during 2024 and 75 days during 2025. The organic loading rate (OLR), calculated as the ratio between the daily influent COD load and the reactor working volume, ranged between approximately 2–5 kg COD m^−3^ d^−1^, with average values of 3 kg COD m^−3^ d^−1^ during stable operating conditions. In parallel, the sludge loading rate based on reactor solids concentration was approximately 0.029–0.031 kg COD kg^−1^ TS d^−1^ over the monitoring period, indicating relatively stable biomass loading conditions despite operational adjustments implemented to optimize UF performance. The membrane UF system operated with a permeate production rate ranging from approximately 0.8 to 1.2 m^3^ h^−1^, while the influent flow to the UF unit was approximately 150 m^3^ h^−1^ and the pressurization pump flow rate was approximately 900 m^3^ h^−1^. The transmembrane pressure (TMP) was generally maintained between 1 and 3 bar. Preliminary sludge filtration was performed using automatic self‐cleaning filters with 0.8 mm mesh size installed on both the feeding and pre‐feeding lines, with automatic impurity discharge cycles of 20 s every 4 min and 1 min 20 s every 5 min, respectively. The reduction of SRT observed in 2025 was intentionally adopted to maintain lower mixed‐liquor solids concentrations, thereby reducing sludge viscosity and membrane fouling propensity, and thus improving long‐term UF stability and energy efficiency.

### 2.4 Removal Yields Calculation

The COD removal efficiency was calculated according to the following equation:

$$\begin{array}{c} \eta_{\mathrm{COD}}\\ =\frac{\left({\mathrm{COD}}_{\text{in}}\times Q_{\text{in}}\right)-\left({\mathrm{COD}}_{\text{permeate}}\times Q_{\text{permeate}}+{\mathrm{COD}}_{\text{waste}}\times Q_{\text{waste}}\right)}{{\mathrm{COD}}_{\text{in}}\times Q_{\text{in}}}\times 100 \end{array}$$

The VS removal efficiency was calculated according to the following equation:

$$\eta_{\mathrm{VS}}=\frac{\left(VS_{\text{in}}\times Q_{\text{in}}\right)-\left(VS_{\text{permeate}}\times Q_{\text{permeate}}+VS_{\text{waste}}\times Q_{\text{waste}}\right)}{VS_{\text{in}}\times Q_{\text{in}}}\times 100$$

### 2.5 Membrane Operation and Cleaning Procedures

The ceramic UF system operated at an average permeate production rate of approximately 1.2 m^3^ h^−1^. Considering the total effective membrane filtration area (0.35 m^2^ per membrane), the average operating filtration flux was approximately 11.5 L m^−2^ h^−1^ (LMH).

To maintain stable hydraulic performance and control membrane fouling, routine physical backwashing was complemented by periodic chemical cleaning‐in‐place (CIP) procedures. Chemical cleaning was performed using a dedicated CIP system consisting of a storage tank, recirculation pumps, and dedicated piping.

The cleaning protocol consisted of three consecutive stages: (i) alkaline cleaning using a sodium hydroxide solution heated to approximately 80°C to remove organic foulants, (ii) acid cleaning using **nitric acid** to dissolve inorganic deposits and scaling, and (iii) a final neutralization/rinsing step before restarting sludge filtration.

The cleaning reagents included 32% sodium hydroxide, 63% nitric acid, and commercial detergent formulations based on surfactants and EDTA (CamClean and Fosfodet).

Chemical cleaning frequency was adjusted according to membrane operating conditions, particularly permeability decline, filtration performance, and evidence of membrane fouling. Under normal operating conditions, a complete chemical cleaning cycle was required approximately every 7–10 days.

### 2.6 Analytical Methods

Chemical–physical analyses were carried out according to Standard Methods (APHA 2012). COD, TS and VS were measured in influent, reactor mixed liquor, and permeate streams. For each parameter, the removal yield, coefficient of variation, standard error of the mean, and 95% confidence interval (normal distribution) were calculated. Table 1 shows the main operating conditions and design parameters of the thermophilic UF‐MBR system.

**TABLE 1:** Main operating conditions and design parameters of the thermophilic UF‐MBR system.

Parameter	Unit of measurement	Value/Range
Wastewater treatment plant capacity	PE	86,400
Reactor configuration	—	Thermophilic pure‐oxygen bioreactor coupled with ceramic UF
Reactor working volume	m^3^	200
Operating temperature	°C	50–55
Oxygen supply	—	Pure oxygen
Influent sludge type	—	Thickened waste activated sludge
Influent flow rate	m^3^ d^−1^	10–30
Hydraulic retention time (HRT)	d	8–12
Solids retention time (SRT)—2024	d	166.7
Solids retention time (SRT)—2025	d	75
Influent COD concentration	mg L^−1^	25,000–40,000
Influent TS concentration	g L^−1^	35–42
Influent VS concentration	g L^−1^	20–30
Organic loading rate (OLR)	kg COD m^−3^ d^−1^	2–5
Sludge loading rate	kg COD kg^−1^ TS d^−1^	0.029–0.031
MLSS concentration	g L^−1^	60–120
MLVSS concentration	g L^−1^	40–60
Membrane type	—	Ceramic ultrafiltration
Number of UF vessels	—	3
Membranes per vessel	—	99
Membrane configuration	—	Tubular
Membrane MWCO	kDa	300
Transmembrane pressure (TMP)	bar	1–4
Retentate management	—	Fully recirculated to reactor
Permeate destination	—	Treated effluent/carbon source recovery
Average filtration flux	L m^−2^ h^−1^	11.5
Chemical cleaning		CIP (alkaline + acid + neutralization)
Cleaning frequency	d	Every 7–10 days (average)
Cleaning reagents		NaOH (32%), HNO_3_ (63%), CamClean, and Fosfodet

The specific sludge production (kg ${\mathrm{VS}}_{\text{produced}}$ kg ${\mathrm{COD}}_{\text{removed}}^{-1}$) was determined as the ratio between the amount of extracted sludge and the COD removed, accounting for biomass accumulation in the reactor throughout the experimental period, following the approach described in the literature (Ma et al. 2012).

## 3 Results

**FIGURE 2:**
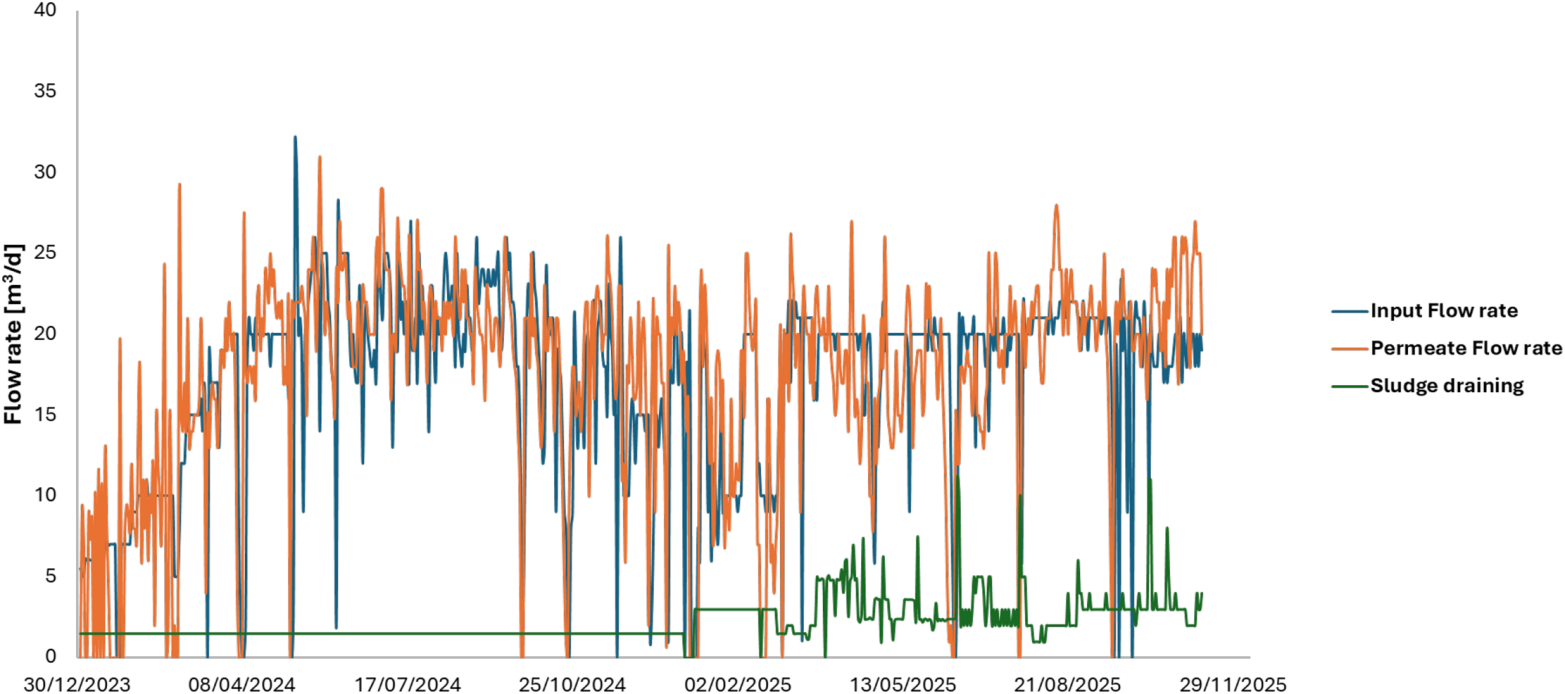
Trends of the influent thickened biological sludge flow, the ultrafiltration permeate flow, and the biological excess sludge flow to control solids concentrations in the thermophilic reactor.

The performance of the thermophilic biological reactor was assessed by analyzing the temporal evolution of the main process parameters and by quantifying removal efficiencies, biomass production, and specific energy and oxygen consumptions. As a preliminary step, Figure 2 illustrates the weekly trends of the thickened biological sludge flow fed to the thermophilic unit, the permeate flow extracted through the UF system, and the excess sludge flow to maintain stable solids concentrations within the reactor. These operational flows provide the baseline for interpreting the subsequent performance indicators. The following subsections present and discuss in detail the results obtained over the entire monitoring period.

### 3.1 COD Evolution and Solids Distribution

**FIGURE 3:**
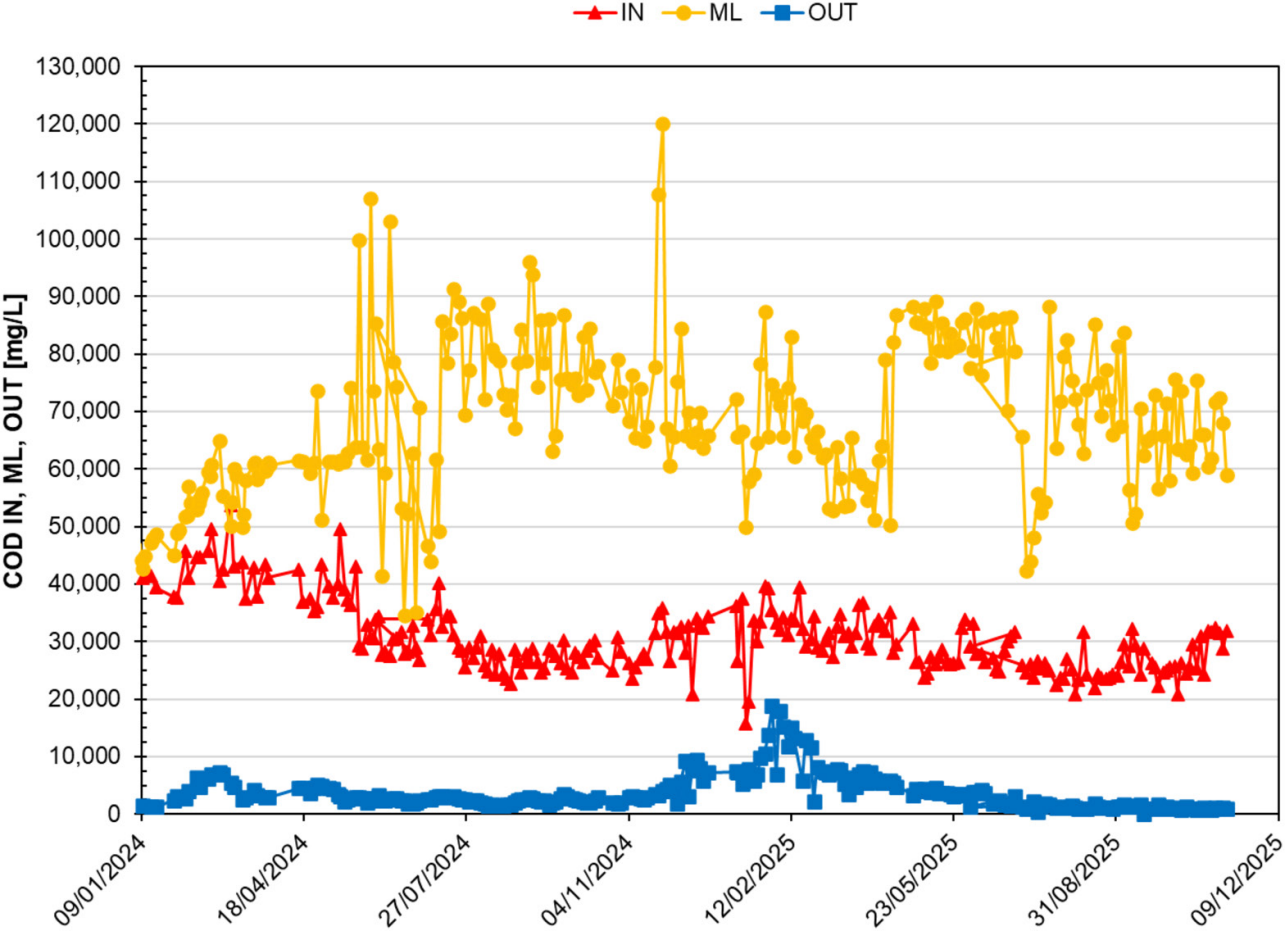
Temporal trends of COD concentrations in the influent thickened sludge (IN), the mixed liquor (ML), and the ultrafiltration permeate (OUT). The stratification between the three streams reflects the active COD biodegradation occurring within the thermophilic reactor and the effective retention of particulate organic matter by the ultrafiltration membrane.

Figure 3 shows the temporal evolution of COD concentrations in the inlet thickened sludge, the mixed liquor, and the UF permeate. The three profiles remain clearly separated throughout the monitoring period, highlighting the specific roles and characteristics of each process stream.

The influent consistently exhibits COD concentrations between 25,000 and 40,000 mg/L, which is typical of thickened sludge with a high organic load. In the thermophilic reactor, the mixed liquor maintains significantly higher COD values, indicating active biochemical conversion under thermophilic conditions and demonstrating stable process performance despite fluctuations in the influent load. This also confirms the effective retention of thermophilic biomass by the UF system, which allows dissolved organic matter to pass through while preventing biomass washout (Aoustin 2001).

The UF membrane's strong selectivity and operational robustness in retaining particulate matter and high‐molecular‐weight organic molecules are demonstrated by the permeate's lowest COD values for the whole duration. Remaining soluble COD in the permeate is a useful resource for downstream uses, such as its reuse as an external carbon source for denitrification, and is compatible with the inherent behavior of membrane processes (Ahmed et al. 2023).

Overall, the results confirm the high biological efficiency of the thermophilic reactor as well as the operational reliability of the membrane separation step.

**FIGURE 4:**
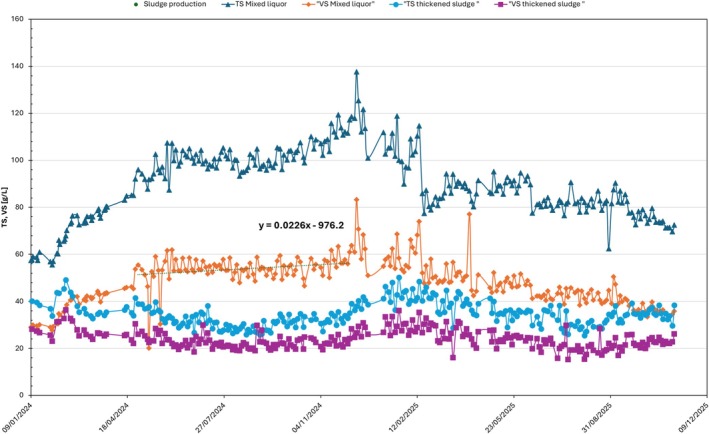
Temporal evolution of total solids (TS) and volatile solids (VS) in the influent and mixed liquor throughout 2024–2025. The figure includes TS and VS measurements for mixed liquor and thickened sludge, as well as sludge production values and the corresponding linear trend.

Figure 4 presents the temporal evolution of TS and VS in the influent and in the mixed liquor. While TS and VS concentrations in the feed exhibited only moderate fluctuations over time, the mixed liquor consistently showed significantly higher solids concentrations. This confirms the ability of the UF system to retain biomass within the bioreactor while allowing only dissolved organic carbon to pass through the membrane. A comparison between TS and VS trends in the mixed liquor highlights the intrinsic biomass yield of thermophilic oxidation processes. The progressive divergence between TS and VS indicates the biodegradation of the volatile (organic) fraction and the simultaneous accumulation of the nonvolatile (inert) fraction. This is evidenced by the more pronounced increase in TS compared to VS within the reactor. Focusing on the periods with maximum data stability, the specific biomass production was estimated at 0.01 kg VS per kg COD removed based on the value of 0.0226 g VS/L·d shown in Figure 4. This value was obtained by analyzing the trend of VS in the oxidation tank during the most stable operating period, calculating the biomass growth coefficient through linear regression, and dividing it by the daily amount of COD removed by the system over the same timeframe, thereby confirming the technology's high capacity for sludge minimization. This value is comparable to, albeit lower than, those reported for other thermophilic biological technologies, which by their nature already show much lower biomass yields than mesophilic ones (Collivignarelli, Carnevale Miino, et al. 2021).

Using the measured TS concentrations, the sludge loading rate was calculated separately for 2024 and 2025, yielding values of 0.0294 and 0.0309 kg COD kg^−1^ TS d^−1^, respectively. These comparable values indicate that the organic load applied to the biomass remained essentially constant between the 2 years. Despite this similarity, the sludge retention time (SRT) differed substantially, decreasing from 166.74 days in 2024 to 75 days in 2025. This reduction was the result of a targeted operational strategy aimed at maintaining lower mixed‐liquor solids concentrations in 2025 to reduce mechanical stress on the UF system, mitigate membrane fouling, improve hydraulic performance, and enhance long‐term membrane stability.

### 3.2 Removal Efficiencies of VS and COD

The removal efficiencies were calculated by comparing the weekly VS loads entering the plant with the corresponding weekly loads leaving the system. The latter were split between the permeate flow from the UF unit and the biological reactor's sludge wastage flow (reported in Figures 3 and 4 as “mixed liquor”), allowing accurate control of in‐reactor concentrations.

It should also be noted that no data are available for January and February 2025, as the plant was temporarily shut down due to the failure of a membrane block. All reported removal efficiencies are expressed on a weekly basis.

**FIGURE 5:**
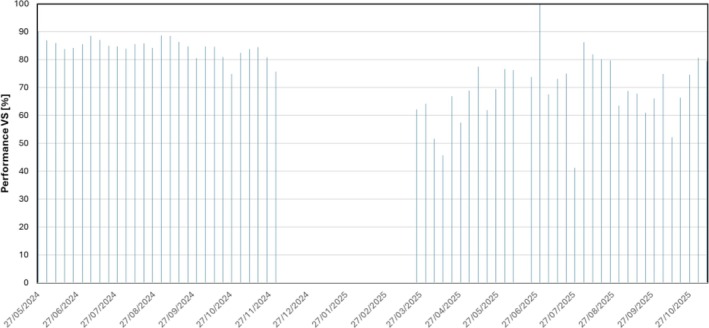
Volatile solids (VS) removal efficiencies over the monitoring period (2024–2025). The data demonstrate the consistently high degradation performance of the thermophilic treatment line, with removals predominantly above 85% and slightly reduced but still substantial performance in 2025.

The VS removal efficiencies (Figure 5) highlight the thermophilic treatment line's exceptional degradation capacity. Throughout much of 2024, the system consistently achieved VS removals approaching or exceeding 85%–90%, with slightly lower but still substantial values observed in 2025. Assuming an average inlet VS concentration of 30 g/L and a treated sludge flowrate of 25 m^3^/d, the 85% VS reduction achieved by the TAMR (Thermophilic Aerobic Membrane Reactor) corresponds to approximately 638 kgVS/d destroyed. This reduction is equivalent to approximately 16 m^3^/d of thickened excess sludge (at ~4% TS) diverted from conventional disposal routes. These results clearly demonstrate the superior volatile‐matter removal performance of thermophilic systems compared to conventional aerobic digestion and even anaerobic digestion, both of which typically exhibit lower degradation rates and significantly longer hydraulic residence times (Wang et al. 2023). The high VS removal underscores the suitability of thermophilic reactors for sludge minimization within advanced wastewater treatment plants.

**FIGURE 6:**
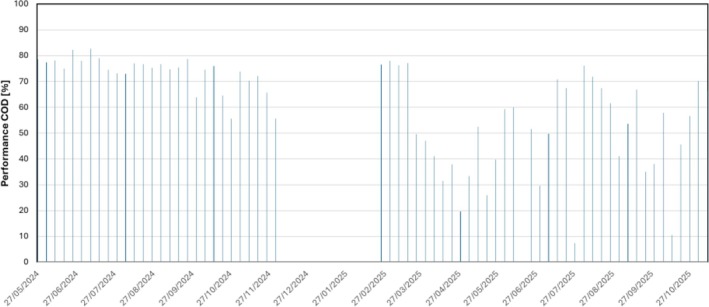
COD removal efficiencies over the monitoring period (2024–2025). The slightly lower removals compared with VS are attributed to the soluble organic fraction passing through the membrane.

Figure 6 shows the COD removal efficiencies, which are slightly lower than those observed for VS. This behavior is expected and is mainly linked to the soluble organic fraction that permeates through the membrane and remains in the treated effluent. Importantly, this characteristic should not be interpreted as a limitation of the thermophilic stage; rather, it represents a functional advantage of the integrated treatment configuration. Previous studies have demonstrated that the COD in the permeate provides an excellent external carbon source for denitrification in the downstream mesophilic biological line (Collivignarelli, Abbà, et al. 2021). As a result, the COD not removed during thermophilic oxidation is effectively recovered and valorized within the overall process chain, highlighting the complementary and synergistic roles of the thermophilic sludge line and the conventional wastewater treatment line (Collivignarelli et al. 2025).

### 3.3 Oxygen‐Based Performance (VS Removal and COD Removal)

**FIGURE 7:**
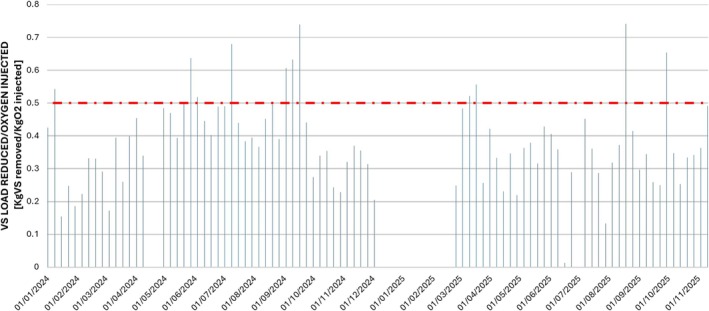
Specific oxygen consumption as a function of volatile solids (VS) removal during the 2024–2025 operational period of the pure‐oxygen thermophilic bioreactor. The system showed stable performance with values clustered around 0.40 kg O_2_ kg^−1^ VS_removed, consistent with literature data for high‐rate thermophilic processes.

The calculated specific oxygen consumption versus VS removal (Figure 7) showed reproducible patterns during the 2024–2025 operating period and is compared to the red dashed line indicating the reference value for this parameter (Tchobanoglous et al. 2003a). Most values clustered around 0.40 ${\mathrm{kg}}_{\mathrm{VS},\text{removed}}$ / ${\mathrm{kg}}_{O_{2},\text{injected}}$, in close agreement with reference literature for high‐rate thermophilic processes (Tchobanoglus et al. 2003b). Moderate variability was observed seasonally, reflecting the temperature‐dependent metabolic dynamics typical of thermophilic biomass (Knapp et al. 2024). Overall, these results confirm the ability of the pure‐oxygen thermophilic bioreactor to sustain elevated oxidation rates while achieving substantial sludge minimization.

**FIGURE 8:**
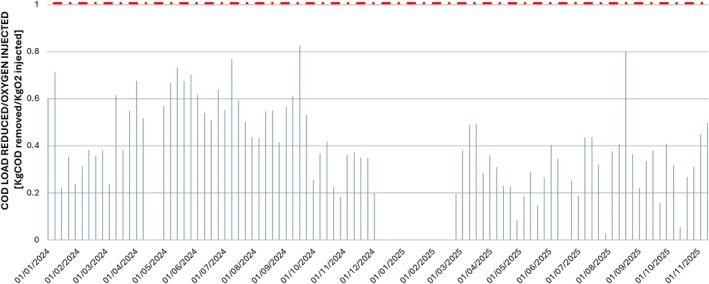
Specific oxygen consumption normalized to COD removal during the 2024–2025 operational period of the thermophilic pure‐oxygen bioreactor. The dashed line indicates the theoretical stoichiometric value.

Lower COD/O_2_ ratios, shown in Figure 8, compared to VS‐based values were observed because a significant portion of soluble COD passed through the UF membranes and was recovered as an external carbon source for downstream denitrification, while only the retained particulate biodegradable COD was oxidized in the thermophilic reactor (Krakkó et al. 2021). The combined interpretation of Figures 3 and 8 clearly highlights the dual functionality of the integrated system: efficient oxidation and minimization of sludge in the thermophilic stage, coupled with the strategic recovery of soluble organic matter to enhance biological nitrogen removal in the denitrification stage.

### 3.4 Energy‐Based Performance (COD Removed per kWh Consumed and VS Removed per kWh Consumed)

**FIGURE 9:**
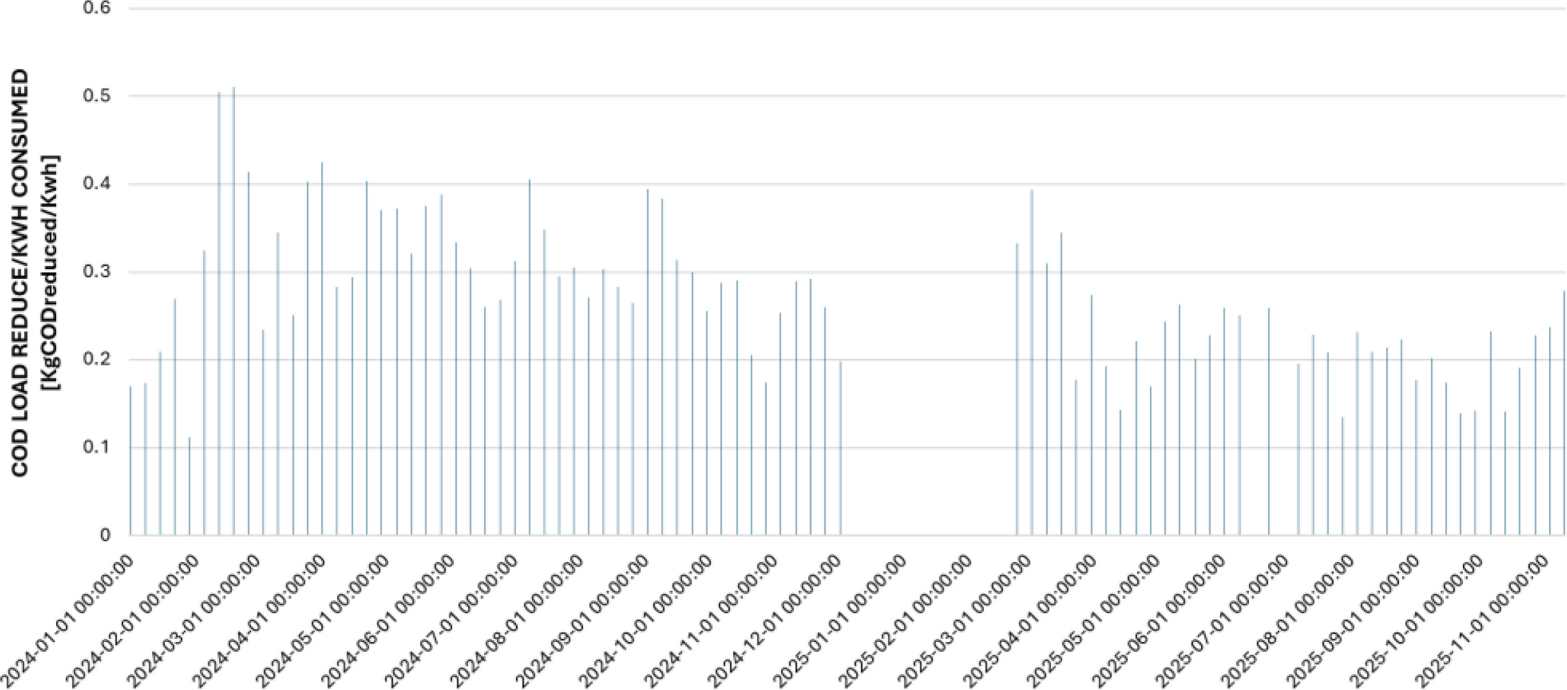
Specific energy consumption expressed as COD removed per unit of electrical energy input during the 2024–2025 operational period.

The specific energy consumption, expressed by normalizing COD removal to the electrical energy input rather than to the supplied oxygen, therefore provides a complementary perspective on the overall process efficiency (Figure 9).

Over the monitoring period, COD removal yields ranged between 0.15 and 0.40 kg COD_removed kWh^−1^.

Higher COD removal per unit of energy was associated with operational phases characterized by stable UF permeability and limited membrane fouling. This confirms the strong dependence of the system's energy efficiency on membrane hydraulic performance. Conversely, operational phases characterized by reduced membrane hydraulic performance, increased fouling, and more frequent backwashing cycles were associated with higher specific energy demands per unit of COD removed.

**FIGURE 10:**
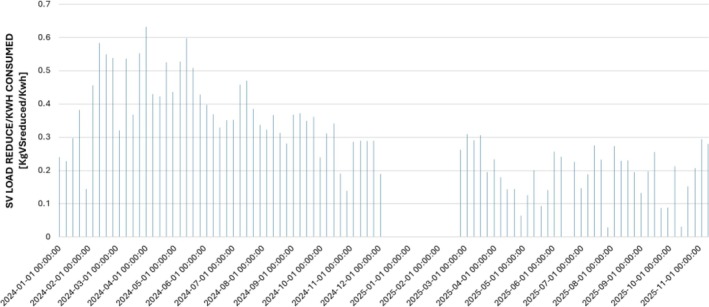
Energy‐based performance of the thermophilic pure‐oxygen biological treatment system, expressed as volatile solids removed per unit of electrical energy consumed.

The energy‐based evaluation of process efficiency was extended by normalizing the electrical energy consumption to the VS removed, providing an additional metric for assessing the biological conversion performance of the thermophilic system (Figure 10). Over the monitoring period, the VS removal per unit of electrical energy input ranged between 0.05 and 0.63 kg VS_removed kWh^−1^, with clear fluctuations associated with changes in membrane and reactor operating conditions.

Periods characterized by high UF permeability, stable fluxes, and limited fouling accumulation were associated with the highest values of VS_removed per kWh, indicating efficient conversion of organic solids into biomass and gaseous end‐products with relatively low energy input. These conditions resulted in a lower amount of VS removed per unit of energy consumed, thereby reducing overall energy efficiency (Collivignarelli et al. 2022).

**FIGURE 11:**
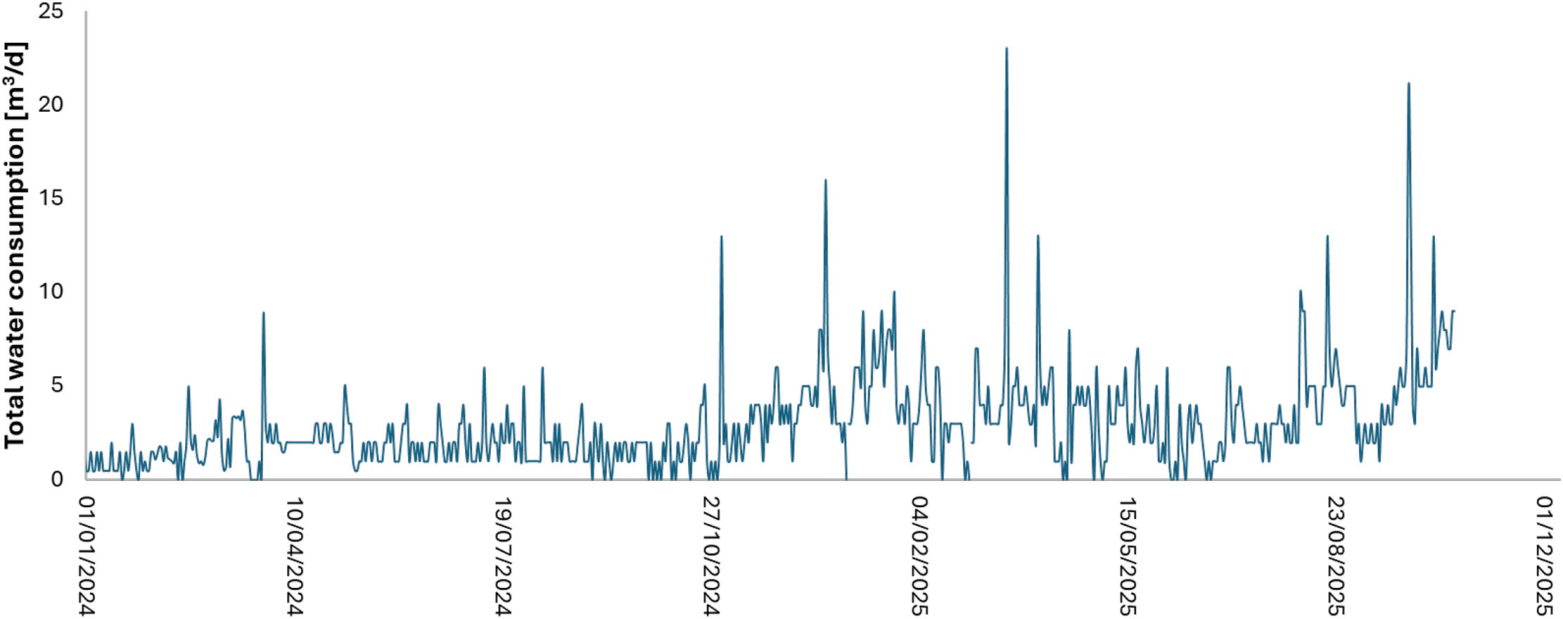
Trend of water consumption associated with membrane backwashing operations over time. The data clearly show an increase in 2025, corresponding to intensified cleaning requirements to maintain membrane performance.

This aspect is confirmed by Figure 11, which illustrates the water consumption associated with the backwashing operations of the membrane block, highlighting a significant increase during 2025. This increase is consistent with the greater frequency and intensity of the backwashing events required to combat fouling and maintain stable filtration conditions.

The results confirm that, similar to the COD‐based indicator, the hydraulic performance of the UF membranes plays a dominant role in determining the energy footprint of the thermophilic process. Operational phases with intensified backwashing, chemical cleanings, or unstable sludge viscosity corresponded to reduced energy performance, highlighting the importance of maintaining optimal membrane operating conditions to sustain favorable energy‐to‐removal ratios.

Since UF represents the primary energy demand of the process, the specific performance values shown in Figures 3 and 4 are particularly relevant for assessing the economic competitiveness of the technology. Although direct comparison between sludge treatment technologies is inherently complex due to differences in sludge characteristics, stabilization targets, solids retention times, and process configurations, literature data generally indicate lower specific energy demands for conventional mesophilic aerobic stabilization processes and anaerobic digestion systems.

Conventional mesophilic aerobic digestion typically exhibits electrical energy consumptions in the range of approximately 0.02–0.08 kWh kg^−1^ TS treated, whereas anaerobic digestion can partially offset energy demand through biogas recovery and cogeneration, often resulting in near energy‐neutral operation under optimized conditions (Tang et al. 2022). In contrast, thermophilic membrane‐based systems generally require higher energy inputs due to elevated aeration demand, heating requirements, recirculation pumping, and membrane filtration constraints, particularly under high‐solids conditions and elevated sludge viscosity (Collivignarelli, Carnevale Miino, et al. 2021; Knapp et al. 2024; Krakkó et al. 2021).

The specific performance observed in the present study (0.15–0.40 kg COD_removed kWh^−1^ and 0.05–0.63 kg VS_removed kWh^−1^) falls within the range reported for advanced thermophilic sludge minimization technologies employing membrane separation and high biomass retention (Collivignarelli, Carnevale Miino, et al. 2021; Knapp et al. 2024). However, unlike conventional stabilization processes, the investigated configuration achieved extremely low biomass yields and VS removals frequently exceeding 85%, while simultaneously allowing recovery of soluble COD in the permeate for downstream denitrification purposes.

Therefore, the higher energy demand associated with the thermophilic UF‐based configuration should be evaluated in relation to the substantial reduction in sludge production and disposal requirements. In regulatory and economic contexts where sludge disposal routes such as agricultural reuse, composting, or landfill applications are increasingly restricted or associated with rising costs, the additional energy demand may be compensated by the reduction in sludge handling, transport, and final disposal costs.

To provide a quantitative assessment of process variability and robustness, descriptive statistical parameters were calculated for the main performance indicators and are reported in Table 2. Mean values, standard deviations (SD), standard errors (SE), coefficients of variation (CV), and 95% confidence intervals (95% CI) were determined for COD removal, VS removal, oxygen utilization efficiency, and energy utilization efficiency. The confidence intervals confirm the overall consistency of process performance throughout the monitoring period, whereas the observed coefficients of variation reflect the operational fluctuations associated with changes in sludge characteristics, membrane maintenance activities, and variable loading conditions. Overall, these statistical indicators support the reliability and reproducibility of the thermophilic UF‐MBR treatment performance discussed above.

**TABLE 2:** Descriptive statistical summary of the main performance indicators of the thermophilic UF‐MBR process during the monitoring period. Mean values, standard deviation (SD), standard error of the mean (SE), coefficient of variation (CV), and 95% confidence intervals (95% CI) are reported.

Parameter	Mean	SD	SE	CV (%)	95% CI
VS removal (%)	65.42	35.52	2.89	54.30	59.70–71.13
COD removal (%)	54.09	35.17	2.82	65.02	48.53–59.65
Specific O_2_ consumption (kg VS removed m^−3^ O_2_)	0.371	0.130	0.013	35.07	0.345–0.398
Specific O_2_ consumption (kg COD removed m^−3^ O_2_)	0.389	0.178	0.018	45.70	0.352–0.425
VS removed per kWh (kg kWh^−1^)	0.271	0.082	0.008	30.24	0.255–0.288
COD removed per kWh (kg kWh^−1^)	0.287	0.134	0.014	46.84	0.260–0.314

### 3.5 Economic Assessment of the Thermophilic UF‐Based Sludge Reduction Process

Although the thermophilic UF‐based sludge minimization process is characterized by higher specific energy demand compared with conventional mesophilic stabilization systems, a preliminary economic evaluation confirms the overall competitiveness of the technology under current sludge management market conditions. The economic performance of the process is mainly influenced by three operational variables: (i) the cost of liquid oxygen supply, (ii) electricity consumption associated primarily with UF and recirculation systems, and (iii) the degree of thickening of the incoming sludge. The organic load of the feed sludge, expressed as COD concentration, is directly related to oxygen demand. For biological sludge, the COD content is approximately 10 kg COD per 1% TS, while the oxygen consumption is approximately 1 kg O_2_ per kg COD removed. Electrical energy demand is instead strongly dependent on sludge concentration and hydraulic loading. For thickened biological sludge with approximately 3% TS in the feed, the average electricity consumption is about 65 kWh m^−3^ of treated sludge. Assuming representative industrial utility costs of 0.12 € kWh^−1^ for electricity and 0.10 € kgO_2_^−1^ for liquid oxygen, the overall specific treatment cost of the thermophilic process is estimated at approximately 70–80 € per ton of sludge dewatered to 18% TS through conventional mechanical dewatering. This value is substantially lower than the current market costs typically associated with sludge disposal via external incineration, which generally range around 250 € per ton including transport and final disposal (Collivignarelli et al. 2025).

These results indicate that, despite the higher operational energy requirements of the thermophilic UF process, the drastic reduction in sludge production and the consequent decrease in disposal demand can compensate for the increased energy expenditure. Furthermore, the system operates with a high degree of automation and remote‐control capability, minimizing labor requirements and reducing the need for dedicated specialized operators. Therefore, the economic competitiveness of the technology should be evaluated considering the entire sludge management chain rather than energy consumption alone, particularly in regulatory contexts where conventional sludge disposal routes are becoming increasingly restricted and expensive.

## 4 Conclusions

This study demonstrates that a full‐scale thermophilic pure‐oxygen process coupled with ceramic UF membranes can reliably achieve very high sludge reduction, with VS removals above 85% and exceptionally low biomass yields. The membrane system ensured stable biomass retention, and the permeation of soluble COD—rather than representing a limitation—provided a valuable carbon source for downstream denitrification.

Process performance and energy efficiency were strongly dependent on UF hydraulic conditions: stable permeability and low fouling corresponded to the highest COD/kWh and VS/kWh values, confirming the central role of membrane behavior in determining operational costs.

Although more energy‐intensive than conventional mesophilic processes, the technology offers a compelling trade‐off in contexts where sludge disposal is costly or restricted. The substantial reduction in biological sludge, combined with improved flexibility and alignment with tightening regulatory frameworks, makes thermophilic UF‐based sludge treatment a robust, competitive, and future‐oriented solution for modern wastewater treatment plants.

## Author Contributions

**Maria Cristina Collivignarelli:** project administration, supervision, resources. **Stefano Bellazzi:** data curation, software, formal analysis, validation, methodology, writing – review and editing, writing – original draft, investigation, conceptualization. **Laura Maria Rita Calabria:** data curation. **Marco Baldi:** writing – review and editing, supervision. **Marco Sordi:** supervision, resources, data curation. **Barbara Marianna Crotti:** writing – review and editing, project administration. **Alessandro Abbà:** data curation, project administration, writing – review and editing, visualization.

## Conflicts of Interest

The authors declare no conflicts of interest.

## Data Availability

The data that support the findings of this study are available on request from the corresponding author. The data are not publicly available due to privacy or ethical restrictions.
